# CD247, a Potential T Cell–Derived Disease Severity and Prognostic Biomarker in Patients With Idiopathic Pulmonary Fibrosis

**DOI:** 10.3389/fimmu.2021.762594

**Published:** 2021-11-22

**Authors:** Yupeng Li, Shibin Chen, Xincheng Li, Xue Wang, Huiwen Li, Shangwei Ning, Hong Chen

**Affiliations:** ^1^ Department of Respiratory and Critical Care Medicine, Second Affiliated Hospital of Harbin Medical University, Harbin, China; ^2^ Medical Research Center, Beijing Chao-Yang Hospital, Capital Medical University, Beijing, China; ^3^ College of Bioinformatics Science and Technology, Harbin Medical University, Harbin, China

**Keywords:** idiopathic pulmonary fibrosis, CD247, immune response, inflammation response, biomarker, prognosis

## Abstract

**Background:**

Idiopathic pulmonary fibrosis (IPF) has high mortality worldwide. The CD247 molecule (CD247, as known as T-cell surface glycoprotein CD3 zeta chain) has been reported as a susceptibility locus in systemic sclerosis, but its correlation with IPF remains unclear.

**Methods:**

Datasets were acquired by researching the Gene Expression Omnibus (GEO). CD247 was identified as the hub gene associated with percent predicted diffusion capacity of the lung for carbon monoxide (Dlco% predicted) and prognosis according to Pearson correlation, logistic regression, and survival analysis.

**Results:**

CD247 is significantly downregulated in patients with IPF compared with controls in both blood and lung tissue samples. Moreover, CD247 is significantly positively associated with Dlco% predicted in blood and lung tissue samples. Patients with low-expression CD247 had shorter transplant-free survival (TFS) time and more composite end-point events (CEP, death, or decline in FVC >10% over a 6-month period) compared with patients with high-expression CD247 (blood). Moreover, in the follow-up 1st, 3rd, 6th, and 12th months, low expression of CD247 was still the risk factor of CEP in the GSE93606 dataset (blood). Thirteen genes were found to interact with CD247 according to the protein–protein interaction network, and the 14 genes including CD247 were associated with the functions of T cells and natural killer (NK) cells such as PD-L1 expression and PD-1 checkpoint pathway and NK cell-mediated cytotoxicity. Furthermore, we also found that a low expression of CD247 might be associated with a lower activity of TIL (tumor-infiltrating lymphocytes), checkpoint, and cytolytic activity and a higher activity of macrophages and neutrophils.

**Conclusion:**

These results imply that CD247 may be a potential T cell-derived disease severity and prognostic biomarker for IPF.

## Introduction

Idiopathic pulmonary fibrosis (IPF) causes worsening dyspnea and deteriorating lung function, which results in poor prognosis (1). Actually, patients with IPF often die within 2–3 years after diagnosis (2, 3), and the 5-year survival rate is less than 40% (4). Therefore, it is important to identify effective biomarkers for disease severity and prognosis in patients with IPF, which might identify patients with a worse predicted prognosis early and might then benefit from more aggressive interventions or earlier referral for transplantation.

Increasing studies have shown that innate and adaptive immune processes can coordinate existing fibrotic responses and are associated with prognosis in patients with IPF (5, 6). CD247 (also referred to as T-cell surface glycoprotein CD3 zeta chain) is part of the T-cell antigen receptor (TCR) complex, playing an important role in receptor expression and signaling (7, 8). Studies have suggested that a low expression of CD247 caused in the setting of chronic inflammation was associated with decreased T cell activity (9–11). Interestingly, the caused immunosuppression is only associated with downregulation of CD247 while the remaining TCR subunits are unaffected, which implies that the CD247 downregulation occurs at chronic inflammation not at acute inflammatory response (9, 10). Furthermore, downregulation of CD247 had been reported in chronic inflammatory diseases such as celiac disease (12), chronic obstructive pulmonary disease (11), systemic lupus erythematosus (13), and systemic sclerosis (14). As far as we know, the association between CD247 and IPF has not been reported.

In this study, according to publicly available databases, we presented evidence of such an association between CD247 and immune microenvironment phenotype and evaluated the role of CD247 expression in patients with IPF.

## Materials and Methods

### Acquisition of Datasets

On the GEO database (http://www.ncbi.nlm.nih.gov/geo/), the datasets meeting the following criteria were included: (1) datasets with data of lung function and (2) datasets with prognostic data (also including the data of decline of lung function). In addition, in order to compare the CD247 expression between IPF and control and clarify the change process of Cd247 expression of T cells in the process of fibrosis, the GSE110147 (15), GSE33566 (16), and GSE141259 (17) datasets (bleomycin-induced mouse model of pulmonary fibrosis) were chosen. In the GSE141259 dataset, single-cell RNA-sequencing (scRNA-seq) data of 0, 3, 7, 10, 14, 21, and 28 days were provided after bleomycin was used. Finally, we selected 11 datasets: four came from lung tissue samples (GSE32537 (18), GSE47460 (19), GSE110147 (15), GSE141259 (17)), six came from blood samples (GSE93606 (20), GSE27957 (21), GSE28042 (21), GSE38958 (22), GSE132607 (23), GSE33566 (16)), and one came from bronchoalveolar lavage fluid (BALF, GSE70866 (24)). Approval of the Ethics Committee was not necessary for the datasets from the GEO database.

### Dataset Preprocessing

The gene expression matrix of GSE32537 (18), GSE47460 (19), GSE110147 (15), GSE93606 (20), GSE28042 (21), GSE38958 (22), GSE132607 (23), GSE33566 (16), and GSE70866 (24) and the raw data of GSE27957 (21) were downloaded from GEO. “Affy” package (25) (v.1.68.0) was used to normalize the array data according to the robust multi-array average (RMA) method (GSE27957). In addition, lung function [percent predicted forced vital capacity (FVC% predicted) and percent predicted diffusion capacity of the lung for carbon monoxide (Dlco% predicted)] was extracted from the GSE93606, GSE38958, GSE132607, GSE32537, and GSE47460 datasets (**Tables 1**, **2**). The data of lung function were not complete in the GSE33566 dataset. Therefore, the data of lung function were not used in this study. Transplant-free survival (TFS) was extracted from the GSE27957 and GSE28042 datasets (**Table 1** **)**. The composite end point (CEP, death, or decline in FVC >10% over a 6-month period) and survival were extracted from the GSE93606 and GSE70866 datasets, respectively (**Tables 1**, **2** and **Supplementary Table 1**). Furthermore, follow-up data were also extracted from the GSE132607 (transcriptome data and lung function data) and GSE93606 datasets (transcriptome data), respectively.

**Table 1:** The clinical features of patients with IPF (blood).

Clinical features	GSE93606	GSE27957	GSE28042	GSE38958	GSE132607	GSE33566
No. of patients	57	45	75	60	74	93
No. of controls	20	0	19	45	0	30
Age (years)	67 ± 8	66.9 ± 8.1	68.9 ± 8.1	68.2 ± 7.2	66.6 ± 7.6	67.2 ± 11.4
Gender						
Male	38 (66.7)	40 (88.9)	52 (69.3)	49 (81.7)	52 (70.3)	61 (65.6)
Female	19 (33.3)	5 (11.1)	23 (30.7)	11 (18.3)	22 (29.7)	32 (34.4)
FVC% predicted	72.22 ± 20.26	62 ± 14	65 ± 16	62.42 ± 14.97	69.66 ± 18.42	–
Dlco% predicted	39.17 ± 14.07	44 ± 17	49 ± 18	43.25 ± 18.73	45.61 ± 15.45	–
Rate of lung transplants	–	2 (4.4)	15 (20)	–	–	–
Immunosuppressive therapy	0	2 (4.4)	11 (14.7)	–	–	0
Status						–
Non-TFS	–	15 (33.3)	43 (57.3)	–	–	–
TFS	–	30 (66.7)	32 (42.7)	–	–	–
CEP*^a^*	34 (59.6)	–	–	–	–	–
Non-CEP	23 (40.4)	–	–	–	–	–
FVC decline ≥10%*^b^*	–	–	–	–	16 (21.6)	–
Dlco decline ≥15%*^b^*	–	–	–	–	26 (35.1)	–

a Death or decline in FVC >10% over the 6-month period.

b Patients experiencing ≥10% relative reduction in FVC% predicted or ≥15% relative reduction in Dlco% predicted over 12 months.

Values are presented as n (%) or mean ± standard deviation (SD).

FVC% predicted, percent predicted forced vital capacity; Dlco% predicted, percent predicted diffusion capacity of the lung for carbon monoxide; TFS, transplant-free survival; CEP, composite end point.

**Table 2:** The clinical features of patients with IPF (lung tissue).

Clinical features	GSE32537	GSE47460	GSE110147
No. of patients	119	122	22
No. of controls	50	91	11
Age (years)	62.6 ± 8.7	64.5 ± 8.4	62 ± 6
Gender			
Male	77 (64.7)	81 (66.4)	17 (77.3)
Female	42 (35.3)	41 (33.6)	5 (22.7)
FVC% predicted	61.25 ± 17.02	64.29 ± 16.42	57 ± 19
Dlco% predicted	45.13 ± 20.30	49.51 ± 18.73	37 ± 10
Immunosuppressive therapy	–	–	15 (68.2)

Values are presented as mean ± SD or n (%).

FVC% predicted, percent predicted forced vital capacity; Dlco% predicted, percent predicted diffusion capacity of the lung for carbon monoxide.

Pearson correlation coefficients were used to determine the association between CD247 and other genes or lung function according to R package “stats” (v.4.0.5) among these datasets. R packages “gplots” (v.3.1.1), “pheatmap” (v.1.0.12), and “RColorBrewer (v.1.1-2)” were used to construct the heatmap. R package “forestplot” (v.1.10.1) was used to construct the forest plot (https://CRAN.R-project.org/package=forestplot).

### Analysis of scRNA-seq Data

The computational analysis of the GSE141259 dataset was performed using R package “Seurat” (4.0.3) (26). Quality control had been finished by the authors of this GSE141259 dataset; therefore, 29,297 cells were analyzed. The Seurat SCTransform () function was used to normalize the scRNA-seq data. Principal component analysis (PCA) was calculated using the Seurat RunPCA () function. UMAP embedding and Louvain clusters were calculated using the first 50 principal components with the Seurat RunUMAP () and FindClusters () functions, respectively. Resolution was set as 0.9. The Seurat FindAllMarkers () function was used to find markers of 31 clusters, and cell types were identified based on markers of each cluster according to the CellMarker (27) and PanglaoDB databases (28). Expression and distribution of Cd247 were visualized according to Seurat DotPlot () and FeaturePlot () functions. Dynamic Cd247 expression was visualized by the web (https://theislab.github.io/LungInjuryRegeneration/).

### The Identification of CD247-Related Partners

We used STRING (http://www.string.embl.de/, version: 11.0 b) (29) to analyze proteins that purportedly interact with CD247 [“medium confidence (0.400)”, meaning of network edges (“evidence”), max number of interactors to show (“no more than 50 interactors” in 1st shell), and active interaction sources (“experiments”)]. The MEM database (30) (https://biit.cs.ut.ee/mem/index.cgi) was used to verify the correlation between CD247 and genes obtained from STRING.

### Functional Analysis

Differentially expressed genes (DEGs) were defined as expression levels of genes that were significantly diverse in IPF patients with low-expression CD247 compared with those with high-expression CD247 (|log Fold Change|>0.5 and false discovery rates (FDR) < 0.05). “Limma” package (v.3.46.0) (31) was used for the analysis of DEGs. Gene Ontology (GO) and Kyoto Encyclopedia of Genes and Genomes (KEGG) were analyzed and visualized according to R package “clusterProfiler” (32). P values were adjusted with the Benjamini and Hochberg (BH) correction method. The single-sample gene set enrichment analysis (ssGSEA) score of 19 immune cells and the activity of 15 immune-related pathways (33) were calculated by the “GSVA” R package (v.1.38.2) (34). CIBERSORT is a large-scale analysis tool of RNA mixtures for cellular biomarkers and therapeutic targets according to the gene expression feature sets of 22 immune cell subtypes (http://cibersort.stanford.edu/) (35). Subsequently, the 20 immune cell subtypes were classified into four types: lymphocytes (B cells naive, B cells memory, plasma cells, T cells CD8, T cells CD4 naive, T cells CD4 memory resting, T cells CD4 memory activated, T cells follicular helper, T cells regulatory, T cells gamma delta, NK cells resting, NK cells activated), macrophages (monocytes, macrophages M0, macrophages M1, macrophages M2), dendritic cells (dendritic cells resting, dendritic cells activated), and mast cells (mast cells resting and mast cells activated).

### Statistical Analysis

SPSS Statistics 23 (IBM SPSS) and R software (Version 4.0.3) were used for statistical analysis. Categorical variables were described as number (%) and were compared by the chi-square test or the Fisher exact test. Continuous variables were compared by independent group t tests. The diagnostic accuracy of CD247 for IPF or Dlco% predicted decline ≥15% over 12 months (Dlco15) were estimated by receiver operating characteristic (ROC) analysis. Youden index was used to calculate the optimal cutoff values of CD247 for Dlco15. Logistic regression analysis was used to estimate the odds ratio (OR) for the statistically significant correlation between gene expression and Dlco15. Kaplan–Meier analysis was used to compare the TFS or CEP or survival between different groups. R package “survminer” (https://CRAN.R-project.org/package=survminer, v.0.4.8) was used to estimate the optimal cutoff expression value of CD247 for the survival analysis. The hazard ratio (HR) for the statistically significant correlation between CD247 expression and TFS or CEP was estimated by univariate Cox regression. The R package “survivalROC” (https://CRAN.R-project.org/package=survivalROC, v.1.0.3) was used to construct the time-dependent ROC curve to evaluate the predictive value of CD247. Some statistical analyses were visualized by GraphPad Prism 9. The bilateral test was used.

## Results

### Identification of the Hub Gene: CD247

**Figure 1:**
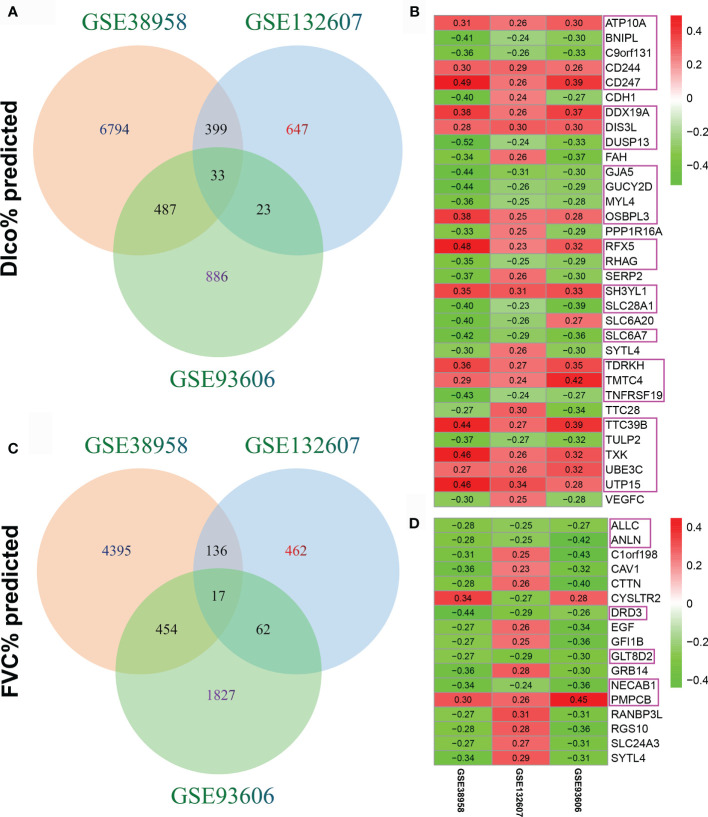
Identification of candidate hub genes. **(A)** The intersection of genes associated with Dlco% predicted in the GSE38958, GSE132607, and GSE93606 datasets. **(B)** Heatmap plot of the 33 genes associated with Dlco% predicted in the three datasets. **(C)** The intersection of genes associated with FVC% predicted in the three datasets. **(D)** Heatmap plot of the 17 genes associated with FVC% predicted in the three datasets. The numbers in the heatmap represent the correlation coefficients, red represents the positive correlation, green represents the negative correlation, the darker shade of red or green represents the higher correlation level, and the pink rectangles showed the genes were selected as the candidate hub genes.

The association between CD247 expression and lung function was respectively determined in the GSE38958, GSE132607, and GSE93606 datasets (blood samples). The significant correlations (p < 0.05) between genes and lung function are shown in **Supplemental Material 1**. After intersecting the genes with significant correlations in the three blood datasets, 33 genes associated with Dlco% predicted and 17 genes associated with FVC% predicted were selected for further analysis (**Supplementary Tables 2**, **3** and **Figure 1**). Furthermore, of the 33 genes, 14 genes with consistent positive correlations (ATP10A, CD244, CD247, DDX19A, DIS3L, OSBPL3, RFX5, SH3YL1, TDRKH, TMTC4, TTC39B, TXK, UBE3C, and UTP15) and 11 genes with consistent negative correlations (BNIPL, C9orf131, DUSP13, GJA5, GUCY2D, MYL4, RHAG, SLC28A1, SLC6A7, TNFRSF19, and TULP2) were selected as the candidate hub genes (**Figure 1B**, pink rectangles). Of the 17 genes, one gene with consistent positive correlations (PMPCB) and five genes with consistent negative correlations (ALLC, ANLN, DRD3, GLT8D2, and NECAB1) were also selected as the candidate hub genes (**Figure 1D**, pink rectangles).

The follow-up data (4, 8, 12 months) were extracted from the GSE132607 dataset (blood). In the fourth month, only MYL4 was significantly negatively associated with Dlco% predicted (**Table 3**). In the eighth month, six genes (CD247, DIS3L, OSBPL3, TDRKH, TTC39B, and UTP15) were significantly positively associated with Dlco% predicted, and three genes (GUCY2D, MYL4, and RHAG) were significantly negatively associated with Dlco% predicted (**Table 3**). In the 12th month, five genes (CD244, CD247, DDX19A, RFX5, and TXK) were significantly positively associated with Dlco% predicted, and MYL4 was significantly negatively associated with Dlco% predicted (**Table 3**). However, no genes were significantly associated with FVC% predicted in the follow-up data. Therefore, CD247 and MYL4 with a greatly consistent significant correlation were chosen for further analysis.

**Table 3:** The correlations between gene expression and Dlco% predicted or FVC% predicted after visiting at 4, 8, and 12 months in the GSE132607 dataset.

Genes	Baseline		4 months		8 months		12 months	
	r	p value	r	p value	r	p value	r	p value
**Dlco% predicted**								
ATP10A	0.258	0.026	0.084	0.484	0.020	0.877	0.011	0.934
BNIPL	-0.239	0.041	0.006	0.960	-0.170	0.175	-0.225	0.083
C9orf131	-0.257	0.027	-0.140	0.242	-0.210	0.094	0.135	0.305
CD244	0.287	0.013	0.114	0.342	0.191	0.127	0.262	0.043
**CD247**	**0.259**	**0.026**	**0.195**	**0.100**	**0.261**	**0.036**	**0.385**	**0.002**
DDX19A	0.257	0.027	0.099	0.406	0.200	0.111	0.271	0.036
DIS3L	0.304	0.008	0.190	0.109	0.324	0.008	0.200	0.126
DUSP13	-0.241	0.039	0.018	0.879	-0.079	0.529	-0.196	0.134
GJA5	-0.308	0.008	-0.102	0.396	-0.196	0.118	-0.018	0.890
GUCY2D	-0.261	0.025	-0.019	0.871	-0.324	0.008	-0.062	0.636
**MYL4**	**-0.254**	**0.029**	**-0.289**	**0.014**	**-0.403**	**0.001**	**-0.433**	**0.001**
OSBPL3	0.250	0.031	0.066	0.581	0.262	0.035	0.142	0.278
RFX5	0.233	0.046	0.185	0.121	0.223	0.074	0.272	0.035
RHAG	-0.250	0.032	-0.221	0.062	-0.247	0.047	-0.233	0.073
SH3YL1	0.315	0.006	0.111	0.352	0.227	0.069	0.199	0.128
SLC28A1	-0.230	0.049	0.069	0.565	-0.014	0.913	-0.089	0.500
SLC6A7	-0.292	0.012	-0.079	0.508	-0.102	0.419	-0.054	0.684
TDRKH	0.265	0.022	0.148	0.215	0.280	0.024	0.118	0.370
TMTC4	0.245	0.036	0.029	0.807	0.004	0.975	-0.058	0.662
TNFRSF19	-0.243	0.037	-0.055	0.644	0.034	0.789	-0.013	0.919
TTC39B	0.272	0.019	0.175	0.141	0.267	0.032	0.209	0.109
TULP2	-0.268	0.021	-0.007	0.955	-0.124	0.324	-0.082	0.533
TXK	0.263	0.024	0.112	0.350	0.171	0.174	0.274	0.034
UBE3C	0.260	0.025	-0.142	0.235	0.220	0.078	-0.093	0.478
UTP15	0.337	0.003	0.087	0.468	0.253	0.042	0.018	0.890
**FVC% predicted**								
ALLC	-0.245	0.036	0.051	0.670	0.031	0.806	0.063	0.630
ANLN	-0.246	0.036	0.109	0.359	0.025	0.838	-0.213	0.103
DRD3	-0.287	0.014	0.005	0.969	0.055	0.659	0.011	0.933
GLT8D2	-0.294	0.012	0.077	0.516	-0.079	0.527	-0.154	0.240
NECAB1	-0.237	0.044	0.146	0.219	0.011	0.928	-0.096	0.466
PMPCB	0.263	0.025	0.010	0.932	-0.102	0.412	-0.123	0.348

Bold values represented that the genes were selected for further study.

**Figure 2:**
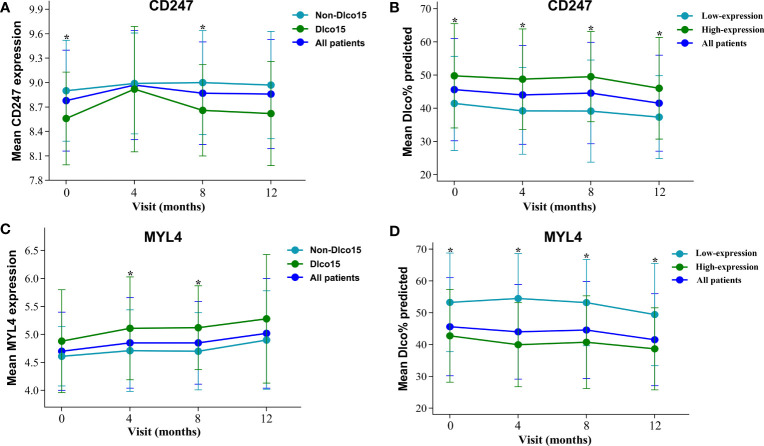
The expression of CD247 and MYL4 and their value for Dlco% predicted after visiting at 0, 4, 8, and 12 months in the GSE132607 dataset. **(A)** The change of mean CD247 expression. **(B)** The comparison of mean Dlco% predicted between high-expression and low-expression CD247. **(C)** The change of mean MYL4 expression. **(D)** The comparison of mean Dlco% predicted between high-expression and low-expression MYL4. *p < 0.05.

**Figure 3:**
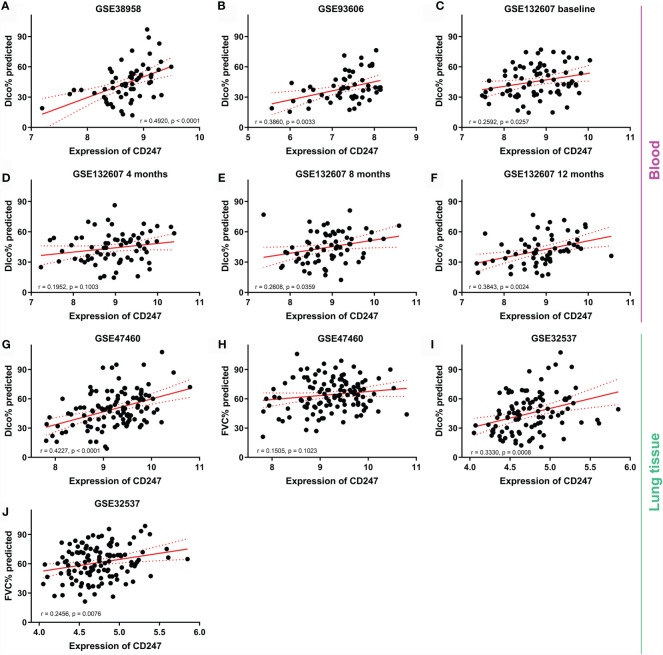
The correlation between CD247 expression and lung function. Blood: GSE38958 dataset **(A)**, GSE93606 dataset **(B)**, visiting at 0, 4, 8, and 12 months in the GSE132607 dataset **(C–F)**. Lung tissue: GSE47460 dataset **(G, H)** and GSE32537 dataset **(I, J)**.

Dlco% predicted decline ≥15% over 12 months (Dlco15) is a useful index to evaluate whether patients with IPF were progressive. Patients with Dlco15 were more likely to have the lower CD247 expression and higher MYL4 expression compared with those without Dlco15 in the GSE132607 dataset (**Figures 2A, C** and **Supplementary Table 4**). In addition, Dlco% predicted in patients with low-expression CD247 classified by the median value of CD247 at baseline was significantly lower than that in patients with high-expression CD247 after visiting at 4, 8, and 12 months in the GSE132607 dataset (**Table 4** and **Figure 2B**). Likewise, Dlco% predicted in patients with high-expression MYL4 classified by the optimal cutoff value (4.37) of MYL4 at baseline was significantly lower than that in patients with low-expression MYL4 after visiting at 4, 8, and 12 months (**Table 4** and **Figure 2D**). Subsequently, two lung tissue datasets (GSE32537 and GSE47460) were used. As shown in **Figure 3**, CD247 expression was significantly positively associated with Dlco% predicted in both blood and lung tissue. However, MYL4 was significantly negatively associated with Dlco% predicted in blood samples, not in lung tissue samples (**Supplementary Figure 1**). Furthermore, CD247 was not significantly correlated with GAP (gender, age, and physiological index) in the GSE70866 dataset (BALF sample, data not shown).

**Table 4:** Baseline characteristics of the patients among different groups in the GSE132607 dataset.

Characteristics	CD247			MYL4		
	High expression	Low expression	p value	High expression	Low expression	p value
Age (years)	66.86 ± 7.32	66.40 ± 8.04	0.796	66.52 ± 7.86	66.93 ± 7.20	0.839
Gender						
Female	8 (21.6)	14 (37.8)	0.127	15 (27.8)	7 (35.0)	0.546
Male	29 (78.4)	23 (62.2)		39 (72.2)	13 (65.0)	
Smoking						
No	12 (32.4)	13 (35.1)	1.000	15 (27.8)	10 (50.0)	0.073
Yes	25 (67.6)	24 (64.9)		39 (72.2)	10 (50.0)	
Baseline						
FVC% predicted	72.14 ± 20.64	67.25 ± 15.9	0.259	67.89 ± 17.8	74.69 ± 19.7	0.168
Dlco% predicted	49.78 ± 15.72	41.44 ± 14.2	0.019	42.76 ± 14.6	53.28 ± 15.5	0.008
4 months						
FVC% predicted	71.62 ± 20.33	65.72 ± 15.9	0.171	66.04 ± 18.4	75.50 ± 16.8	0.049
Dlco% predicted	48.78 ± 15.14	39.23 ± 13.1	0.006	39.97 ± 13.2	54.51 ± 14.1	< 0.001
8 months						
FVC% predicted	72.31 ± 20.35	65.35 ± 15.3	0.118	65.82 ± 18.2	76.07 ± 16.8	0.034
Dlco% predicted	49.51 ± 13.58	39.16 ± 15.4	0.006	40.73 ± 14.6	53.21 ± 13.6	0.002
12 months						
FVC% predicted	68.63 ± 21.55	64.36 ± 14.5	0.375	64.94 ± 18.0	70.49 ± 18.8	0.301
Dlco% predicted	46.02 ± 15.32	37.33 ± 12.5	0.019	38.65 ± 12.9	49.46 ± 16.1	0.009

**Figure 4:**
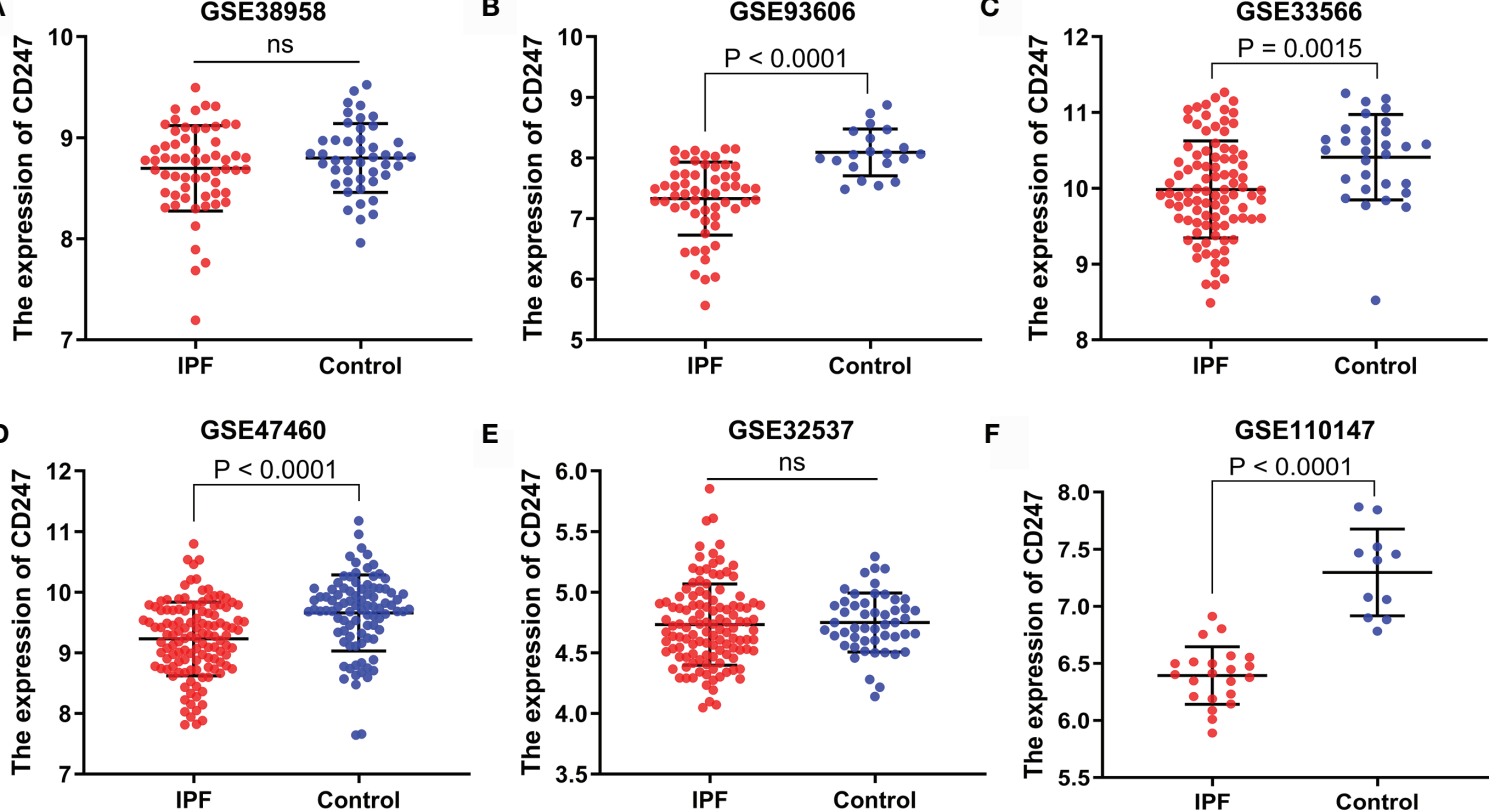
The expression of CD247 in both blood and lung tissue. Blood: GSE38958 dataset **(A)**, GSE93606 dataset **(B)**, GSE33566 dataset **(C)**. Lung tissue: GSE47460 dataset **(D)**, GSE32537 dataset **(E)**, GSE110147 dataset **(F)**. ns, not significant.

The expression of CD247 in patients with IPF was lower than that in controls in the GSE93606 (blood), GSE33566 (blood), GSE47460 (lung tissue), and GSE110147 (lung tissue) datasets, whereas the expression of CD247 did not show a difference in the GSE38958 (blood) and GSE32537 (lung tissue) datasets (**Figure 4**). The expression of CD247 in patients with IPF (n = 75) was lower than that in controls (n = 19) in the GSE28042 dataset (13.24 vs. 13.56, p = 0.0043). Immunosuppressive therapy was not used before the blood samples were collected in the GSE93606 and GSE33566 datasets, implying that the expression of CD247 in the blood samples was not affected by the immunosuppressive therapy (**Table 1**). The diagnostic values of CD247 for IPF were variable in different datasets (**Supplementary Figure 2**). Besides, the expression of MYL4 did not show the significant difference in these datasets (data not shown). Therefore, CD247 was considered as the key gene.

### The Prognostic Value of CD247 in the Blood Samples

**Figure 5:**
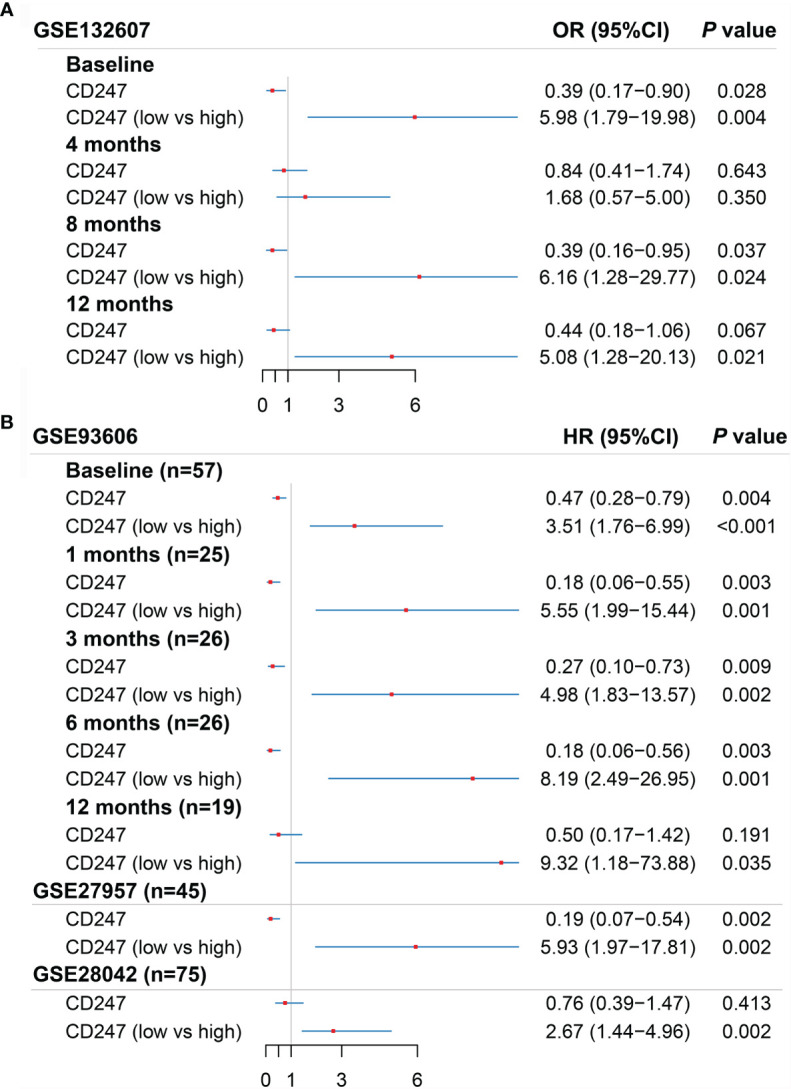
Results of the univariate logistic regression regarding Dlco15 in the GSE132607 dataset **(A)** and the univariate Cox regression regarding CEP in the GSE93606 dataset and non-TFS in the GSE27957 and GSE28042 datasets **(B)**. Dlco15, Dlco% predicted decline ≥15% over 12 months; TFS, non-transplant-free survival; CEP, composite end point (death or decline in FVC >10% over six months period).

According to the ROC curve analysis and R package “survminer”, the optimal cutoff value of CD247 was chosen for the logistic regression analysis in the GSE132607 (blood) dataset and prognosis-related analysis in the GSE93606 (blood), GSE27957 (blood), and GSE28042 (blood) datasets, respectively. According to logistic regression analysis, a low expression of CD247 at visiting at 0, 8, and 12 months was the risk factor of Dlco15 in the GSE132607 dataset, whereas the low expression of CD247 was not the risk factor of Dlco15 in the fourth month (**Figure 5A**). According to Cox regression analysis and Kaplan–Meier analysis, low-expression CD247 at visiting at 0, 1, 3, 6, and 12 months was the risk factor of CEP in the GSE93606 dataset and significantly associated with shorter TFS time in the GSE27957 and GSE28042 datasets (**Figure 5B**, **Supplementary Figures 3A–G**). Furthermore, the ROC curve showed that the areas under the curve (AUC) were 0.736 at 1 year and 0.741 at 2 years for CEP in the GSE93606 dataset (**Supplementary Figure 3H**). Moreover, AUCs for non-TFS were respectively 0.889, 0.787, and 0.702 at 1, 2, and 3 years in the GSE27957 dataset, whereas the AUC was relatively low in the GSE28042 dataset (**Supplementary Figures 3I, J**). In the GSE70866 dataset (BALF samples), CD247 was not significantly associated with mortality (p > 0.05, **Supplementary Table 5**).

### Functional Analysis

**Figure 6:**
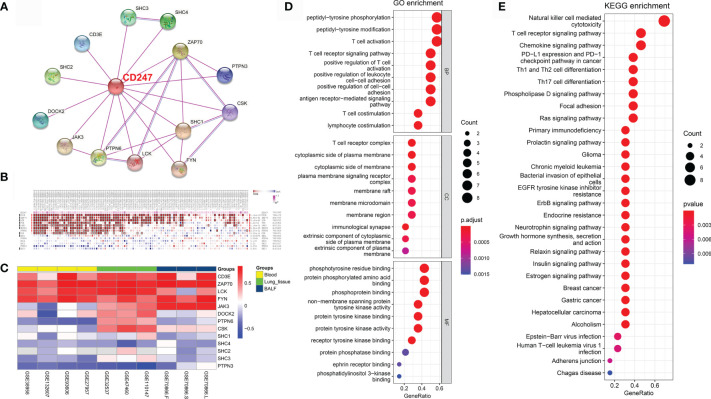
CD247-related gene enrichment analysis. **(A)** The available experimentally determined CD247-binding proteins using the STRING tool. **(B)** According to the MEM database, the co-expression correlations between CD247 and 13 selected genes were analyzed. **(C)** The corresponding Pearson coefficients of the 13 genes correlated with CD247 were visualized by the heatmap in the blood, lung tissue, and BALF samples. The darker shade of red or blue represents the higher correlation level. **(D)** The top 10 significant terms in the biological processes (BP), cellular component (CC), and molecular function (MF) analysis for the 14 genes. **(E)** The top 30 significant terms in the KEGG pathways analysis for the 14 genes.

The protein–protein interaction (PPI) network was constructed based on CD247 according to the STRING database (average node degree: 3.71, PPI enrichment p-value: <1.62e^-3^; **Figure 6A**) (29). According to the PPI network, 13 protein-coding genes (CD3E, SHC2, DOCK2, JAK3, PTPN6, LCK, FYN, SHC1, CSK, PTPN3, ZAP70, SHC4, and SHC3) were found to interact with CD247. The MEM database (30) gathers several hundreds of publicly available gene expression data sets from the ArrayExpress database, which is a useful tool for the co-expression analysis across hundreds of datasets. Results from the MEM database (30) revealed a significant co-expression relationship between CD247 and the 9 of 13 interacted genes (CD3E, ZAP70, LCK, FYN, JAK3, DOCK2, PTPN6, CSK, and SHC1; **Figure 6B**). In addition, in patients with IPF, results from blood, lung tissue, and BALF samples revealed a significant co-expression association between CD247 and the 6 of 13 interacted genes (CD3E, ZAP70, LCK, FYN, JAK3, and PTPN3; **Figure 6C**). GO and KEGG analysis implied that CD247 and its 13 interacted genes were enriched in the immune response especially the T cell-related biological processes (BP), cellular component (CC), and pathways (**Figures 6D, E** **)**. Furthermore, CD247, ZAP70, LCK, FYN, PTPN6, SHC1, SHC4, SHC2, and SHC3 were associated with NK cell-mediated cytotoxicity (**Supplementary Table 6**). Interestingly, five genes (CD247, CD3E, ZAP70, LCK, PTPN6) were associated with the hsa05235 pathway (PD-L1 expression and PD-1 checkpoint pathway in cancer, **Supplementary Table 6**).

**Figure 7:**
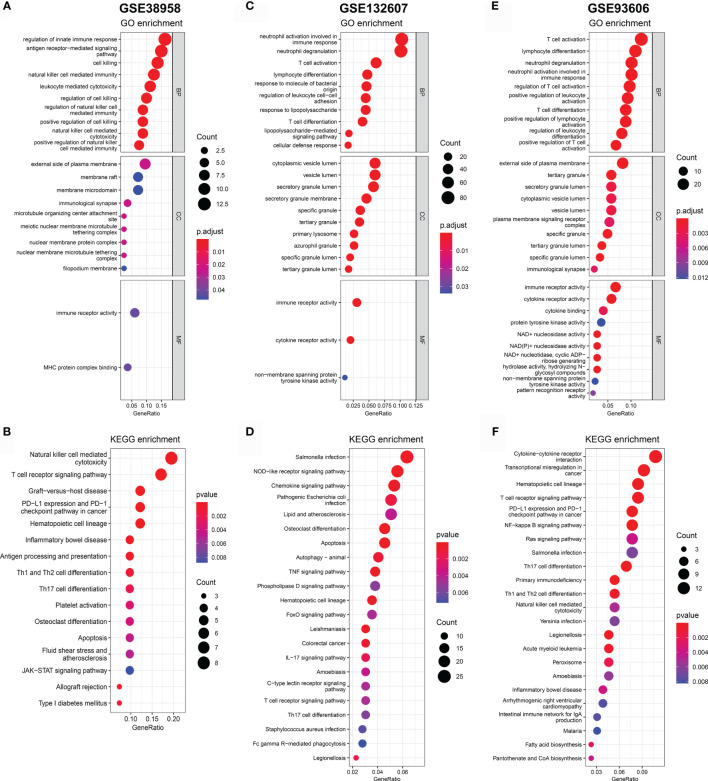
Significant GO terms and KEGG pathway analysis for the DEGs between patients with low-expression CD247 and patients with high-expression CD247. The most significant GO enrichment and KEGG pathways in the GSE38958 dataset **(A, B)**, GSE132607 dataset **(C, D)**, and GSE93606 datasets **(E, F)** are displayed.

**Figure 8:**
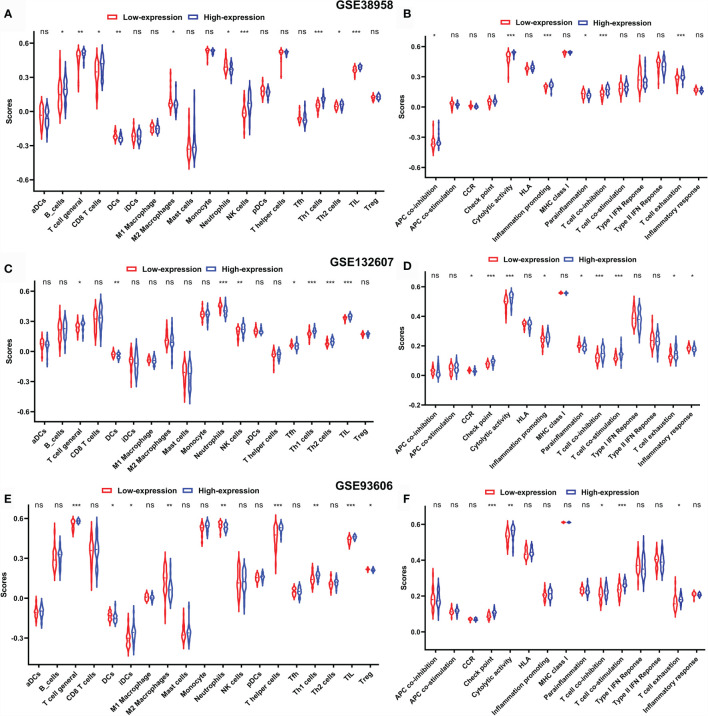
Comparison of the ssGSEA scores between patients with low-expression CD247 and patients with high-expression CD247 in the GSE38958 dataset **(A, B)**, GSE132607 dataset **(C, D)**, and GSE93606 dataset **(E, F)**. The scores of 19 immune cells **(A, C, E)** and 15 immune-related functions **(B, D, F)** are displayed in violin plots. DC, dendritic cell; TIL, tumor-infiltrating lymphocytes; CCR, cytokine–cytokine receptor. P values were shown as: ns, not significant; *p < 0.05; **p < 0.01; ***p < 0.001.

In order to reveal the biological significance correlated with CD247, the DEGs between the patients with high-expression and low-expression CD247 based on the median value were used to conduct the GO enrichment and KEGG pathway analysis in the GSE38958, GSE132607, and GSE93606 datasets (blood samples). In the three datasets, DEGs were mainly enriched in inflammation- and immune-related response and pathways such as neutrophil activation involved in immune response, T cell activation, T cell differentiation, leukocyte chemotaxis, IL-17 signaling pathway, T cell receptor signaling pathway, Th17 cell differentiation, PD-L1 expression, and PD-1 checkpoint pathway in cancer and so on (**Figure 7**). Furthermore, related functions or pathways with ssGSEA showed that patients with low-expression CD247 were more likely to have lower immune activity [lower T cell general, Th1 cells, TIL (tumor-infiltrating lymphocytes), checkpoint, cytolytic activity, T cell co-inhibition, and T cell exhaustion scores)] and higher degree of inflammation response [higher DCs (dendritic cell), M2 macrophages, and neutrophils] compared with patients with high-expression CD247 in the three datasets (**Figure 8**), which was consistent with the results of CIBERSORT analysis (**Supplementary Figures 4A, C, E**). In addition, low-expression CD247 was significantly associated with low lymphocyte score (**Supplementary Figures 4B, D, F**).

### The scRNA-seq Data Analysis of CD247

According to the LungMAP database (36), CD247 is mainly expressed by the T cells and NK cells in the human lung based on scRNA-seq data (37) (**Supplementary Figure 5A**). In the mouse lung, Cd247 is also mainly expressed by the T cells and NK cells, and the expression of Cd247 of T cells is increased in the acute inflammatory stage, then decreased in the fibrotic stage after bleomycin injection (**Supplementary Figures 5B–D**).

## Discussion

IPF is a serious lung disease with high mortality. In this study, we observed a significantly downregulated CD247 expression in patients with IPF compared with controls, and CD247 was significantly positively associated with Dlco% predicted in both blood and lung samples. The significantly positive association was still consistent after following up 8 and 12 months in the GSE132607 dataset (blood). Besides, low-expression CD247 was also a risk factor of Dlco15 in the GSE132607 dataset (blood) and is significantly associated with higher CEP in the GSE93606 datasets (blood). After following up at 1, 3, 6, and 12 months, low-expression CD247 was still the risk factor of CEP in the GSE93606 dataset (blood). In addition, low expression of CD247 was significantly associated with shorter TFS time in the GSE27957 and GSE28042 datasets (blood). The cause of low AUC in the GSE28042 dataset might be the high rate of lung transplantation. Therefore, CD247 might be a potential biomarker for disease severity and prognosis in patients with IPF. In addition, MYL4 was significantly negatively associated with Dlco% predicted in blood samples whereas it was associated neither with Dlco% predicted in the lung tissue samples nor with prognosis in the blood samples. Thereby, MYL4 might be an effective biomarker of disease severity for IPF in blood samples. However, the roles of the two genes need further study for verification.

CD247 as part of the TCR complex plays an important role in receptor expression and signaling (7, 8) and is associated with chronic inflammation (9). According to the LungMAP database, CD247 is mainly expressed by the T cells and NK cells in the lung based on scRNA-seq data, implying that CD247 could be an important regulator of immune responses in the lung. Actually, inflammation caused by some pathogens such as viruses and bacteria are proposed to play a role in the development of IPF. In the bleomycin-induced pulmonary fibrosis model, cytomegalovirus is considered to accelerate existing fibrosis according to enhancing TGFB1 activation and the expression of both phospho-SMAD2 and Vimentin (38). Besides, in BALF and lung tissue of patients with IPF, Epstein–Barr virus (EBV, a member of the Herpes family) is enriched compared with healthy controls (39, 40). Furthermore, GERD (gastroesophageal reflux disease) is common in patients with IPF; thereby, the ongoing micro aspiration could lead to repeated inoculation with oral and gastric organisms (41).

In this study, results from blood, lung tissue, and BALF samples revealed significant co-expression associations between CD247 and the six interacted genes (CD3E, ZAP70, LCK, FYN, JAK3, and PTPN3). When the expression of CD247 was downregulated, the expressions of CD3E, ZAP70, LCK, and FYN were also downregulated. JAK3 was negatively associated with CD247 in the blood samples but was positively associated with CD247 in the lung tissue and BALF samples. These genes are key signaling molecules not only in the selection and maturation of developing T-cells but also in the activation of T cells. These results reveal that downregulation of CD247 caused by inflammation might suppress the immune activity by regulating the expression of these genes, which needs further study for verification.

In addition, according to the GO and KEGG analyses of DEGs between the patients with low-expression and high-expression CD247, these DEGs were mainly enriched in inflammation- and immune-related response and pathways. Besides, patients with low-expression CD247 were more likely to have lower activity of T cells in general, Th1 cells, NK cells, and TIL (tumor-infiltrating lymphocytes) and higher activity of dendritic cells (DCs), M2 macrophages, and neutrophils compared with patients with high-expression CD247. Furthermore, the response process analysis showed that patients with low-expression CD247 were more likely to have a lower score of checkpoint, cytolytic activity, and T cell activation and higher score of inflammation compared with patients with high-expression CD247. These results were consistent with the following studies.

The incidence of cancer in IPF patients is higher compared with matched controls, especially for lung cancer (42). Interestingly, five genes (CD247, CD3E, ZAP70, LCK, PTPN6) and DEGs between the patients with high-expression and low-expression CD247 were associated with the hsa05235 pathway (PD-L1 expression and PD-1 checkpoint pathway in cancer), and patients with low-expression CD247 had a lower TIL ssGSEA score, which may explain the high incidence of cancer. Besides, the downregulation of T cell regulatory genes associated with the immune checkpoint CTLA-4 was significantly associated with reduced event-free survival in the PBMCs (peripheral blood mononuclear cell) of patients with IPF (21). Interestingly, inflammatory interstitial lung diseases were caused by checkpoint inhibitors used as cancer immunotherapy (43). We speculated that the low immune checkpoint expression may be associated with the development and progression of IPF, which needs further study for verification.

Immune processes can coordinate existing fibrotic responses and are associated with prognosis in patients with IPF (5, 6). Th1 cells and their secretory products such as IL-12 (a potent inducer of IFN-γ) are considered as being anti-fibrotic (44, 45). Macrophages play an important role in the pathogenesis of IPF according to the regulation of both injury and repair of lung (46, 47). A relative excess of M1/M2 macrophages leads to epithelial cell death as well as aberrant and dysregulated repair responses, which could cause progression or acute exacerbation of IPF (48, 49). Neutrophils are associated with production of cytokines and chemokines, tissue injury, regulation of ECM (extracellular matrix) turnover, and generation of NETs (neutrophil extracellular traps), which result in fibroblast activation and ECM accumulation in IPF (6).

Taken together, our results suggest that chronic inflammation could participate in the development and progression of IPF according to the downregulated expression of CD247.

There are several limitations in this study. First, the study was conducted based on the retrospective data from GEO, and the number of samples in each dataset was relatively small. Second, we have only considered a single variable in the logistic regression and Cox regression. Many prominent prognostic clinical parameters such as lung function, treatment measures, and underlying diseases were not reported in most datasets that we used; thereby, the prognostic value of CD247 was limited. Third, the treatment of patients with IPF was unknown in some datasets, which may affect both disease and data analysis. Fourth, results regarding Dlco15 and CEP were based on a single dataset, which limited the generalizability of these findings. Finally, larger-sample prospective studies are needed to estimate the clinical relevance of CD247.

## Conclusion

These results suggest that CD247 could reflect well the immune activity in both lung and blood and may be a potential biomarker to predict the lung function and prognosis of patients with IPF. Besides, MYL4 may be a potential biomarker for Dlco% predicted in the blood samples. However, the results need further study for verification.

## Data Availability Statement

Publicly available datasets were analyzed in this study. This data can be found here: GSE32537 (https://www.ncbi.nlm.nih.gov/geo/query/acc.cgi?acc=GSE32537), GSE47460 (https://www.ncbi.nlm.nih.gov/geo/query/acc.cgi?acc=GSE47460), GSE110147 (https://www.ncbi.nlm.nih.gov/geo/query/acc.cgi?acc=GSE110147), GSE141259 (https://www.ncbi.nlm.nih.gov/geo/query/acc.cgi?acc=GSE141259), GSE93606 (https://www.ncbi.nlm.nih.gov/geo/query/acc.cgi?acc=GSE93606, GSE27957 (https://www.ncbi.nlm.nih.gov/geo/query/acc.cgi?acc=GSE27957), GSE28042 (https://www.ncbi.nlm.nih.gov/geo/query/acc.cgi?acc=GSE28042), GSE38958 (https://www.ncbi.nlm.nih.gov/geo/query/acc.cgi?acc=GSE38958), GSE132607 (https://www.ncbi.nlm.nih.gov/geo/query/acc.cgi?acc=GSE132607), GSE33566 (https://www.ncbi.nlm.nih.gov/geo/query/acc.cgi?acc=GSE33566), GSE70866 (https://www.ncbi.nlm.nih.gov/geo/query/acc.cgi?acc=GSE70866). The annotated gene set file used in ssGSEA were available in **Supplementary Material 2**.

## Ethics Statement

Approval of the Ethics Committee was not necessary for the datasets from GEO database.

## Author Contributions

HL, XL, and XW performed the data analysis. YL and SC performed the data collection, prepared the first manuscript draft, validated the data collection, refined the research idea, performed the data analysis, and edited the manuscripts. HC and SN designed and developed the research idea, refined the research idea, validated the data collection, and edited the manuscripts. HC and SN are the guarantors of the manuscript. All authors contributed to the article and approved the submitted version.

## Conflict of Interest

The authors declare that the research was conducted in the absence of any commercial or financial relationships that could be construed as a potential conflict of interest.

## Publisher’s Note

All claims expressed in this article are solely those of the authors and do not necessarily represent those of their affiliated organizations, or those of the publisher, the editors and the reviewers. Any product that may be evaluated in this article, or claim that may be made by its manufacturer, is not guaranteed or endorsed by the publisher.

## Supplementary Material

Supplementary Material 1 The all significantly correlations with FVC% predicted and Dlco% predicted in the GSE38958, GSE132607, and GSE93606 datasets.

Supplementary Material 2 The annotated gene set file used in ssGSEA.

Supplementary Figure 1 The correlation between MYL4 expression and lung function. Blood: GSE38958 dataset **(A)**, GSE93606 dataset **(B)**, visiting 0, 4, 8, 12 months in the GSE132607 dataset **(C–F)**. Lung tissue: GSE47460 dataset **(G, H)** and GSE32537 dataset **(I, J)**.

Supplementary Figure 2 The diagnostic value of CD247 for IPF according to ROC curve. Blood: GSE38958 dataset **(A)**, GSE93606 dataset **(B)**, GSE33566 dataset **(C)**. Lung tissue: GSE47460 dataset **(D)**, GSE32537 dataset **(E)**, GSE110147 dataset **(F)**.

Supplementary Figure 3 Survival analyses based on the optimal cut-off expression value of CD247 in the GSE93606 **(A-E)**, GSE27957 **(F)**, and GSE28042 **(G)** datasets. Time-dependent ROC curves for the predictive value of CD247 in the GSE93606 **(H)**, GSE27957 **(I)**, and GSE28042 **(J)** datasets.

Supplementary Figure 4 Comparison of the CIBERSORT scores between patients with low-expression CD247 and patients with high-expression CD247 in the GSE38958 dataset **(A, B)**, GSE132607 dataset **(C, D)** and GSE93606 dataset **(E, F)**. The scores of 22 immune cells **(A, C, E)** and 4 main immune cell types **(B, D, F)** are displayed in box plots. P values were showed as: ns, not significant; *P < 0.05; **P < 0.01; ***P < 0.001; ****P < 0.0001.

Supplementary Figure 5 The scRNA-seq data analysis. **(A)** lungMAP database (https://www.lungmap.net/), **(B–D)** the mouse lung (GSE141259 dataset).

## Abbreviations

IPF, idiopathic pulmonary fibrosis; CD247, CD247 molecule; GEO, Gene Expression Omnibus; FVC% predicted, percent predicted forced vital capacity; Dlco% predicted, percent predicted diffusion capacity of the lung for carbon monoxide; TFS, transplant-free survival; CEP, composite end point; NK, natural killer; TIL, tumor-infiltrating lymphocytes; TCR, T-cell antigen receptor; scRNA-seq, single-cell RNA-sequencing; BALF, bronchoalveolar lavage fluid; RMA, robust multi-array average; Dlco15, Dlco% predicted decline ≥ 15% over 12 months; DEGs, differentially expressed genes; GO, gene ontology; KEGG, Kyoto Encyclopedia of Genes and Genomes; ssGSEA, single-sample gene set enrichment analysis; SD, standard deviation; ROC, receiver operating characteristic; HR, hazard ratio; AUC, area under the curves; GAP, gender, age, and physiological index; PPI, protein–protein interaction; BP, biological processes; CC, cellular component; MF, molecular function; EBV, Epstein–Barr virus; DCs, dendritic cells; ECM, extracellular matrix.
