# Supplementary figures and images for: CD247, a Potential T Cell–Derived Disease Severity and Prognostic Biomarker in Patients With Idiopathic Pulmonary Fibrosis
Source: Front Immunol. 2021 Nov 22;12:762594. doi: 10.3389/fimmu.2021.762594 (PMC8645971; doi:10.3389/fimmu.2021.762594)

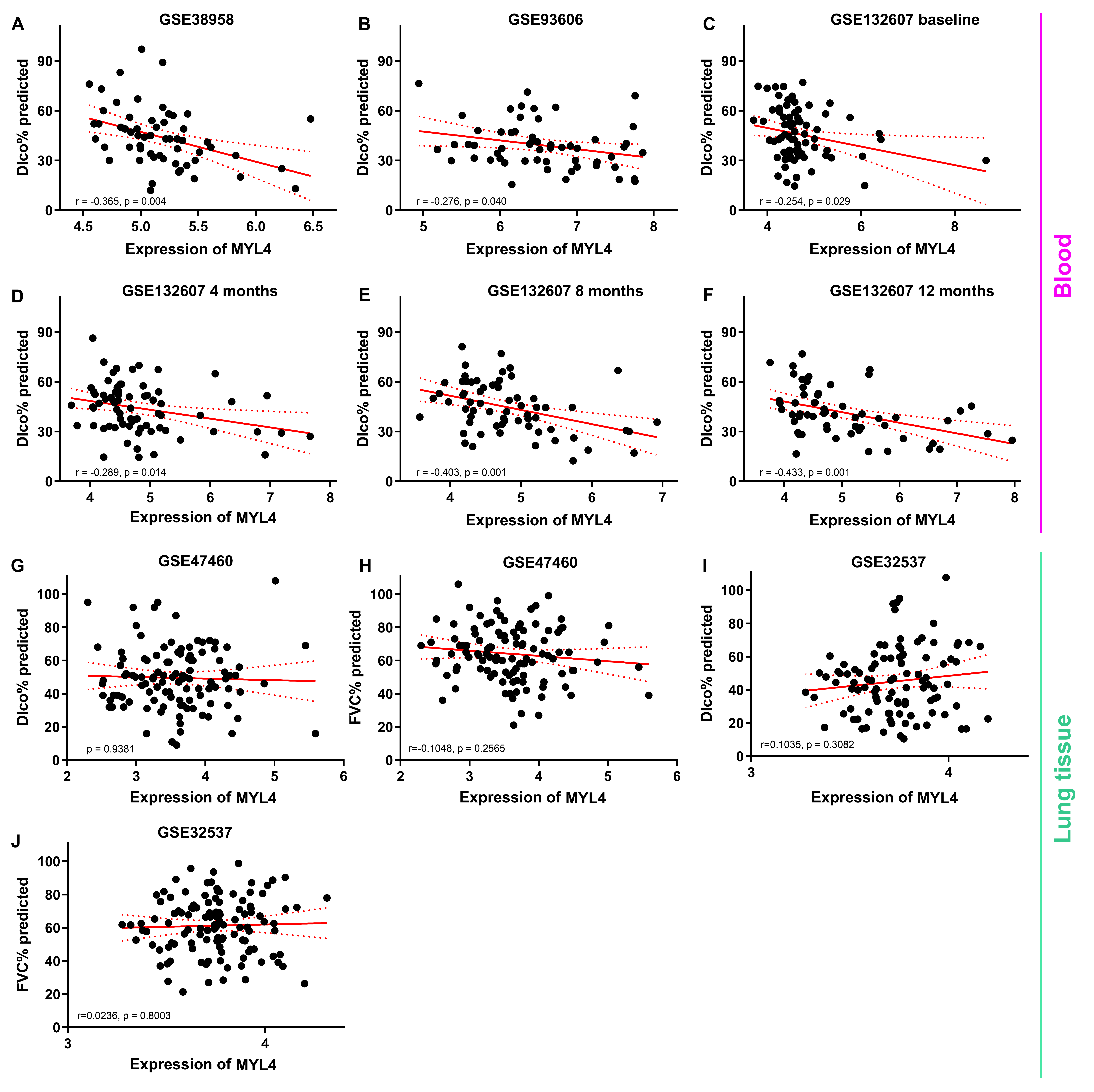

Supplement: Supplementary Figure 1 — The correlation between MYL4 expression and lung function. Blood: GSE38958 dataset (A), GSE93606 dataset (B), visiting 0, 4, 8, 12 months in the GSE132607 dataset (C–F). Lung tissue: GSE47460 dataset (G, H) and GSE32537 dataset (I, J). [file Image_1.tif]

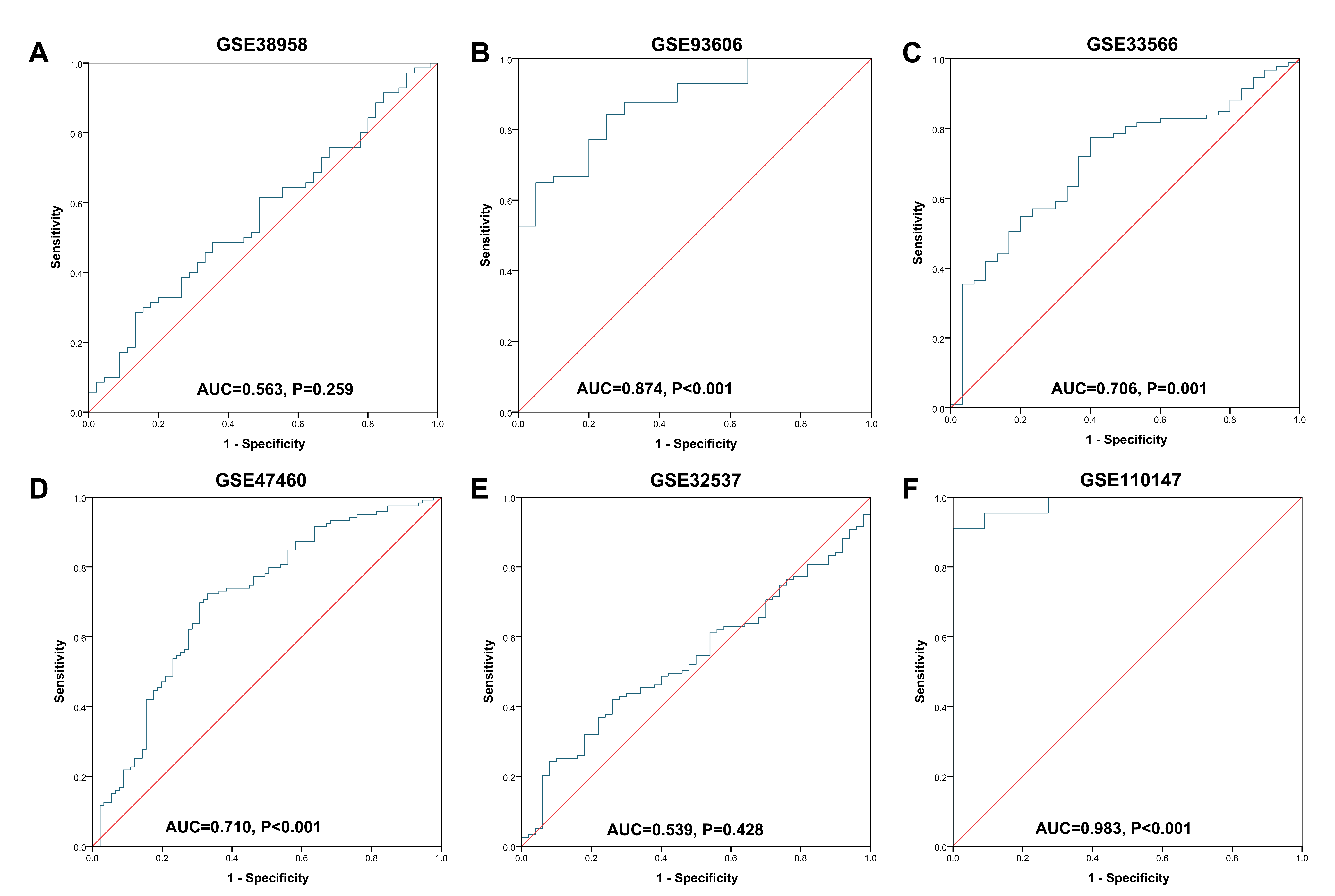

Supplement: Supplementary Figure 2 — The diagnostic value of CD247 for IPF according to ROC curve. Blood: GSE38958 dataset (A), GSE93606 dataset (B), GSE33566 dataset (C). Lung tissue: GSE47460 dataset (D), GSE32537 dataset (E), GSE110147 dataset (F). [file Image_2.tif]

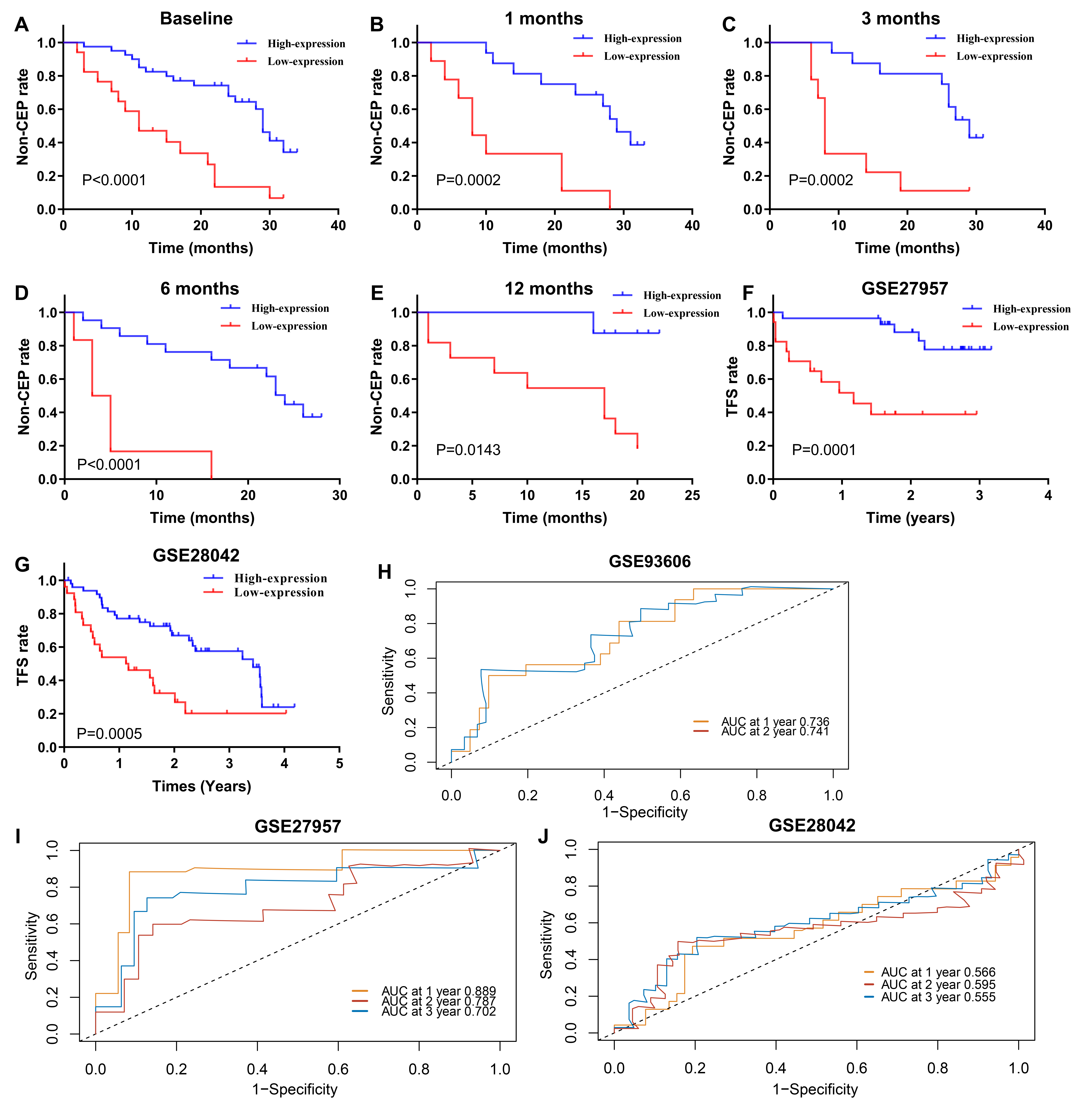

Supplement: Supplementary Figure 3 — Survival analyses based on the optimal cut-off expression value of CD247 in the GSE93606 (A-E), GSE27957 (F), and GSE28042 (G) datasets. Time-dependent ROC curves for the predictive value of CD247 in the GSE93606 (H), GSE27957 (I), and GSE28042 (J) datasets. [file Image_3.tif]

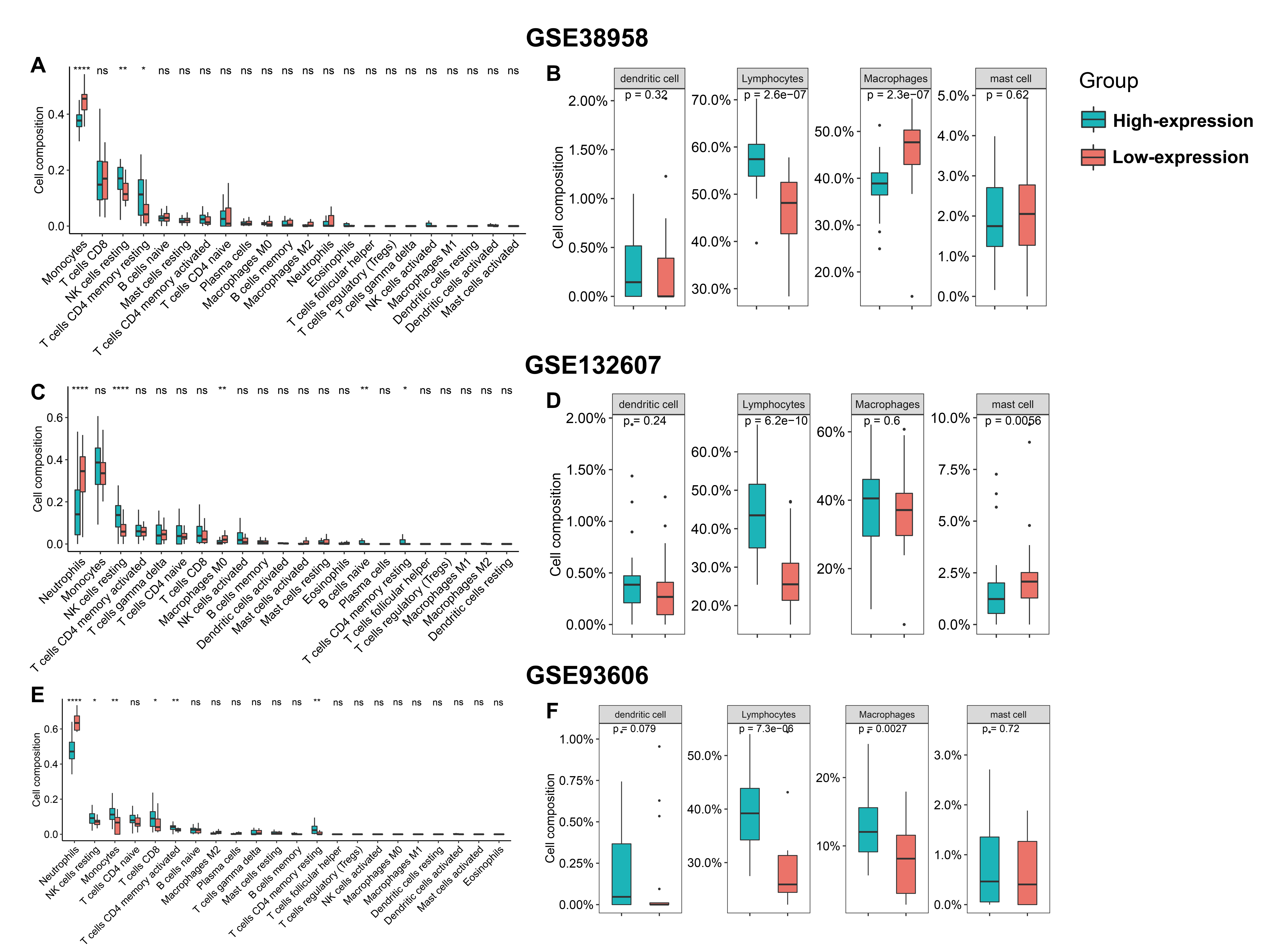

Supplement: Supplementary Figure 4 — Comparison of the CIBERSORT scores between patients with low-expression CD247 and patients with high-expression CD247 in the GSE38958 dataset (A, B), GSE132607 dataset (C, D) and GSE93606 dataset (E, F). The scores of 22 immune cells (A, C, E) and 4 main immune cell types (B, D, F) are displayed in box plots. P values were showed as: ns, not significant; *P < 0.05; **P < 0.01; ***P < 0.001; ****P < 0.0001. [file Image_4.tif]

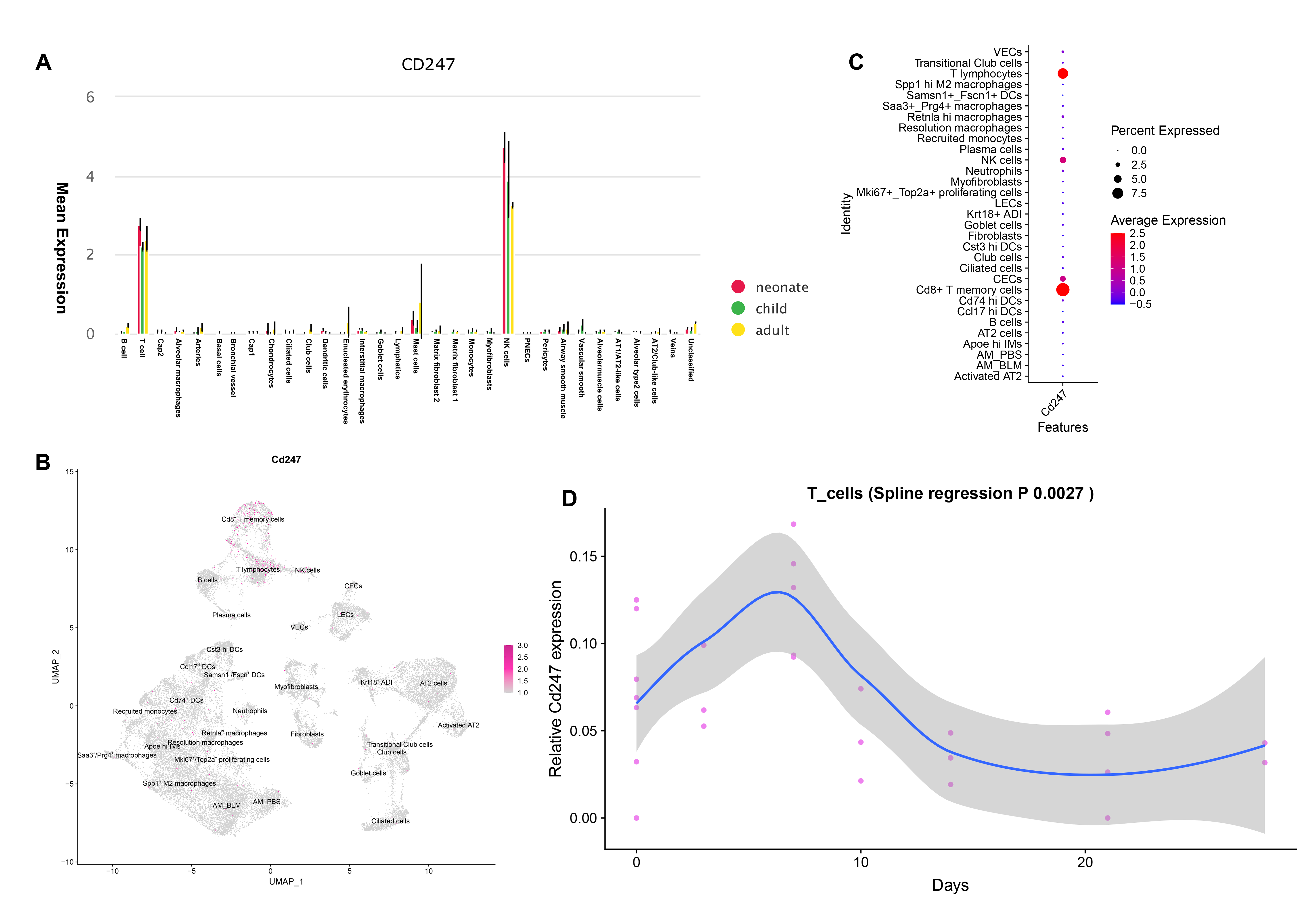

Supplement: Supplementary Figure 5 — The scRNA-seq data analysis. (A) lungMAP database (https://www.lungmap.net/), (B–D) the mouse lung (GSE141259 dataset). [file Image_5.tif]
