# Supplementary material for: CD247, a Potential T Cell–Derived Disease Severity and Prognostic Biomarker in Patients With Idiopathic Pulmonary Fibrosis
Source: Front Immunol. 2021 Nov 22;12:762594. doi: 10.3389/fimmu.2021.762594 (PMC8645971; doi:10.3389/fimmu.2021.762594)
Supplement: Supplementary file 8 [file Table_1.docx]

Table S1. The clinical features of patients with IPF in the GSE70866 dataset (BALF).

Values are presented as mean ± SD or n (%).

| Clinical features | **Freiburg** | **SIENA** | **LEUVEN** |
| --- | --- | --- | --- |
| No. of patients | 62 | 50 | 64 |
| No. of controls | 20 | 0 | 0 |
| Age (years) | 67.4 ± 9.1 | 68.7 ± 11.2 | 68.3 ± 8.5 |
| Gender |  |  |  |
| Male | 53 (85.5) | 40 (80.0) | 51 (79.7) |
| Female | 9 (14.5) | 10 (20.0) | 13 (20.3) |
| GAP | 4.47 ± 1.64 | 4.62 ± 1.82 | 3.81 ± 1.42 |
| Immunosuppressive therapy | 0 | 0 | 0 |
| Status |  |  |  |
| Alive | 17 (27.4) | 19 (38.0) | 40 (62.5) |
| Dead | 45 (72.6) | 31 (62.0) | 24 (37.5) |
