# Supplementary material for: CD247, a Potential T Cell–Derived Disease Severity and Prognostic Biomarker in Patients With Idiopathic Pulmonary Fibrosis
Source: Front Immunol. 2021 Nov 22;12:762594. doi: 10.3389/fimmu.2021.762594 (PMC8645971; doi:10.3389/fimmu.2021.762594)
Supplement: Supplementary file 9 [file Table_2.docx]

**Table S2**. The correlation between genes expression and Dlco% predicted.

| Genes | GSE38958 |  |  | GSE132607 |  |  | GSE93606 |  |
| --- | --- | --- | --- | --- | --- | --- | --- | --- |
|  | r | P value |  | r | P value |  | r | P value |
| **ATP10A** | **0.315** | **0.014** |  | **0.258** | **0.026** |  | **0.303** | **0.023** |
| **BNIPL** | **-0.408** | **0.001** |  | **-0.239** | **0.041** |  | **-0.298** | **0.026** |
| **C9orf131** | **-0.361** | **0.005** |  | **-0.257** | **0.027** |  | **-0.332** | **0.012** |
| **CD244** | **0.297** | **0.021** |  | **0.287** | **0.013** |  | **0.264** | **0.049** |
| **CD247** | **0.492** | **0.000** |  | **0.259** | **0.026** |  | **0.386** | **0.003** |
| CDH1 | -0.403 | 0.001 |  | 0.236 | 0.043 |  | -0.273 | 0.042 |
| **DDX19A** | **0.376** | **0.003** |  | **0.257** | **0.027** |  | **0.368** | **0.005** |
| **DIS3L** | **0.282** | **0.029** |  | **0.304** | **0.008** |  | **0.296** | **0.027** |
| **DUSP13** | **-0.517** | **0.000** |  | **-0.241** | **0.039** |  | **-0.325** | **0.014** |
| FAH | -0.343 | 0.007 |  | 0.264 | 0.023 |  | -0.373 | 0.005 |
| **GJA5** | **-0.441** | **0.000** |  | **-0.308** | **0.008** |  | **-0.299** | **0.025** |
| **GUCY2D** | **-0.444** | **0.000** |  | **-0.261** | **0.025** |  | **-0.289** | **0.031** |
| **MYL4** | **-0.365** | **0.004** |  | **-0.254** | **0.029** |  | **-0.276** | **0.040** |
| **OSBPL3** | **0.381** | **0.003** |  | **0.250** | **0.031** |  | **0.279** | **0.038** |
| PPP1R16A | -0.330 | 0.010 |  | 0.247 | 0.034 |  | -0.289 | 0.031 |
| **RFX5** | **0.476** | **0.000** |  | **0.233** | **0.046** |  | **0.315** | **0.018** |
| **RHAG** | **-0.352** | **0.006** |  | **-0.250** | **0.032** |  | **-0.285** | **0.033** |
| SERP2 | -0.372 | 0.003 |  | 0.260 | 0.025 |  | -0.298 | 0.025 |
| **SH3YL1** | **0.353** | **0.006** |  | **0.315** | **0.006** |  | **0.332** | **0.012** |
| **SLC28A1** | **-0.403** | **0.001** |  | **-0.230** | **0.049** |  | **-0.388** | **0.003** |
| SLC6A20 | -0.397 | 0.002 |  | -0.256 | 0.027 |  | 0.269 | 0.045 |
| **SLC6A7** | **-0.424** | **0.001** |  | **-0.292** | **0.012** |  | **-0.362** | **0.006** |
| SYTL4 | -0.298 | 0.021 |  | 0.257 | 0.027 |  | -0.299 | 0.025 |
| **TDRKH** | **0.360** | **0.005** |  | **0.265** | **0.022** |  | **0.350** | **0.008** |
| **TMTC4** | **0.288** | **0.026** |  | **0.245** | **0.036** |  | **0.422** | **0.001** |
| **TNFRSF19** | **-0.428** | **0.001** |  | **-0.243** | **0.037** |  | **-0.272** | **0.042** |
| TTC28 | -0.268 | 0.039 |  | 0.297 | 0.010 |  | -0.337 | 0.011 |
| **TTC39B** | **0.440** | **0.000** |  | **0.272** | **0.019** |  | **0.392** | **0.003** |
| **TULP2** | **-0.375** | **0.003** |  | **-0.268** | **0.021** |  | **-0.319** | **0.016** |
| **TXK** | **0.455** | **0.000** |  | **0.263** | **0.024** |  | **0.324** | **0.015** |
| **UBE3C** | **0.271** | **0.036** |  | **0.260** | **0.025** |  | **0.324** | **0.015** |
| **UTP15** | **0.459** | **0.000** |  | **0.337** | **0.003** |  | **0.278** | **0.038** |
| VEGFC | -0.302 | 0.019 |  | 0.253 | 0.029 |  | -0.280 | 0.037 |
