# Supplementary material for: CD247, a Potential T Cell–Derived Disease Severity and Prognostic Biomarker in Patients With Idiopathic Pulmonary Fibrosis
Source: Front Immunol. 2021 Nov 22;12:762594. doi: 10.3389/fimmu.2021.762594 (PMC8645971; doi:10.3389/fimmu.2021.762594)
Supplement: Supplementary file 10 [file Table_3.docx]

**Table S3**. The correlation between genes expression and FVC% predicted.

| Genes | GSE38958 |  |  | GSE132607 |  |  | GSE93606 |  |
| --- | --- | --- | --- | --- | --- | --- | --- | --- |
|  | r | P value |  | r | P value |  | r | P value |
| **ALLC** | **-0.279** | **0.031** |  | **-0.245** | **0.036** |  | **-0.270** | **0.043** |
| **ANLN** | **-0.282** | **0.029** |  | **-0.246** | **0.036** |  | **-0.424** | **0.001** |
| C1orf198 | -0.312 | 0.015 |  | 0.252 | 0.031 |  | -0.425 | 0.001 |
| CAV1 | -0.357 | 0.005 |  | 0.231 | 0.050 |  | -0.317 | 0.016 |
| CTTN | -0.281 | 0.030 |  | 0.259 | 0.027 |  | -0.403 | 0.002 |
| CYSLTR2 | 0.335 | 0.009 |  | -0.266 | 0.023 |  | 0.276 | 0.038 |
| **DRD3** | **-0.440** | **0.000** |  | **-0.287** | **0.014** |  | **-0.261** | **0.0497** |
| EGF | -0.273 | 0.035 |  | 0.257 | 0.028 |  | -0.343 | 0.009 |
| GFI1B | -0.268 | 0.038 |  | 0.249 | 0.033 |  | -0.361 | 0.006 |
| **GLT8D2** | **-0.271** | **0.036** |  | **-0.294** | **0.012** |  | **-0.297** | **0.025** |
| GRB14 | -0.356 | 0.005 |  | 0.283 | 0.015 |  | -0.297 | 0.025 |
| **NECAB1** | **-0.339** | **0.008** |  | **-0.237** | **0.044** |  | **-0.356** | **0.007** |
| **PMPCB** | **0.299** | **0.020** |  | **0.263** | **0.025** |  | **0.446** | **0.001** |
| RANBP3L | -0.268 | 0.039 |  | 0.312 | 0.007 |  | -0.305 | 0.021 |
| RGS10 | -0.282 | 0.029 |  | 0.282 | 0.015 |  | -0.356 | 0.007 |
| SLC24A3 | -0.275 | 0.034 |  | 0.267 | 0.023 |  | -0.313 | 0.018 |
| SYTL4 | -0.339 | 0.008 |  | 0.291 | 0.013 |  | -0.306 | 0.021 |
