# Supplementary material for: CD247, a Potential T Cell–Derived Disease Severity and Prognostic Biomarker in Patients With Idiopathic Pulmonary Fibrosis
Source: Front Immunol. 2021 Nov 22;12:762594. doi: 10.3389/fimmu.2021.762594 (PMC8645971; doi:10.3389/fimmu.2021.762594)
Supplement: Supplementary file 11 [file Table_4.docx]

**Table S4.** The changes of CD247 and MYL4 expression after visiting 0, 4, 8, 12 months in the GSE132607 dataset.

| **Groups** | CD247 | P value |  | MYL4 | P value |
| --- | --- | --- | --- | --- | --- |
| **0 month (n=74)** | 8.78 ± 0.62 |  |  | 4.70 ± 0.70 |  |
| Dlco15 | 8.56 ± 0.57 | 0.023 |  | 4.88 ± 0.92 | 0.119 |
| Non-Dlco15 | 8.90 ± 0.62 |  |  | 4.61 ± 0.53 |  |
| **4 months (n=74)** | 8.97 ± 0.67 |  |  | 4.85 ± 0.81 |  |
| Dlco15 | 8.92 ± 0.77 | 0.648 |  | 5.11 ± 0.92 | 0.048 |
| Non-Dlco15 | 8.99 ± 0.62 |  |  | 4.71 ± 0.73 |  |
| **8 months (n=68)** | 8.87 ± 0.63 |  |  | 4.85 ± 0.74 |  |
| Dlco15 | 8.66 ± 0.56 | 0.031 |  | 5.12 ± 0.75 | 0.022 |
| Non-Dlco15 | 9.00 ± 0.64 |  |  | 4.70 ± 0.69 |  |
| **12 months (n=60)** | 8.86 ± 0.67 |  |  | 5.02 ± 0.98 |  |
| Dlco15 | 8.62 ± 0.64 | 0.061 |  | 5.28 ± 1.15 | 0.158 |
| Non-Dlco15 | 8.97 ± 0.66 |  |  | 4.90 ± 0.88 |  |
