# Supplementary material for: CD247, a Potential T Cell–Derived Disease Severity and Prognostic Biomarker in Patients With Idiopathic Pulmonary Fibrosis
Source: Front Immunol. 2021 Nov 22;12:762594. doi: 10.3389/fimmu.2021.762594 (PMC8645971; doi:10.3389/fimmu.2021.762594)
Supplement: Supplementary file 12 [file Table_5.docx]

**Table S5.** The predicted value of CD247 for death in the GSE70866 dataset.

| **Groups** | HR (95%CI) | P value |
| --- | --- | --- |
| Freiburg | 1.11 (0.92-1.35) | 0.285 |
| SIENA | 1.07 (0.79-1.45) | 0.677 |
| LEUVEN | 1.19 (0.92-1.54) | 0.192 |
